# Analysis of Arc/Arg3.1 Oligomerization In Vitro and in Living Cells

**DOI:** 10.3390/ijms25126454

**Published:** 2024-06-12

**Authors:** Barbara Barylko, Clinton A. Taylor, Jason Wang, Per Niklas Hedde, Yan Chen, Kwang-Ho Hur, Derk D. Binns, Chad A. Brautigam, George N. DeMartino, Joachim D. Mueller, David M. Jameson, Joseph P. Albanesi

**Affiliations:** 1 Department of Pharmacology, University of Texas Southwestern Medical Center, 6001 Forest Park, Dallas, TX 75390, USA; barbara.barylko@utsouthwestern.edu (B.B.); clinton.taylor@utsouthwestern.edu (C.A.T.4th); derk.binns@utsouthwestern.edu (D.D.B.); 2 Department of Physiology, University of Texas Southwestern Medical Center, 6001 Forest Park, Dallas, TX 75390, USA; jason.wang2@utsouthwestern.edu (J.W.); george.demartino@utsouthwestern.edu (G.N.D.); 3 Department of Cell and Molecular Biology, John A. Burns School of Medicine, University of Hawaii, 651 Ilalo St., BSB 222, Honolulu, HI 96813, USA; phedde@uci.edu; 4 Laboratory for Fluorescence Dynamics, University of California, Irvine, CA 92697, USA; 5 School of Physics and Astronomy, University of Minnesota, Minneapolis, MN 55455, USA; chenx238@umn.edu (Y.C.); hurxx018@umn.edu (K.-H.H.); jochen@umn.edu (J.D.M.); 6 Department of Biophysics, University of Texas Southwestern Medical Center, 6001 Forest Park, Dallas, TX 75390, USA; chad.brautigam@utsouthwestern.edu

**Keywords:** activity-regulated-cytoskeleton-associated protein, Arc/Arg3.1, oligomerization, fluorescence fluctuation spectroscopy, Förster resonance energy transfer, fluorescence lifetime imaging

## Abstract

Arc (also known as Arg3.1) is an activity-dependent immediate early gene product enriched in neuronal dendrites. Arc plays essential roles in long-term potentiation, long-term depression, and synaptic scaling. Although its mechanisms of action in these forms of synaptic plasticity are not completely well established, the activities of Arc include the remodeling of the actin cytoskeleton, the facilitation of AMPA receptor (AMPAR) endocytosis, and the regulation of the transcription of AMPAR subunits. In addition, Arc has sequence and structural similarity to retroviral Gag proteins and self-associates into virus-like particles that encapsulate mRNA and perhaps other cargo for intercellular transport. Each of these activities is likely to be influenced by Arc’s reversible self-association into multiple oligomeric species. Here, we used mass photometry to show that Arc exists predominantly as monomers, dimers, and trimers at approximately 20 nM concentration in vitro. Fluorescence fluctuation spectroscopy revealed that Arc is almost exclusively present as low-order (monomer to tetramer) oligomers in the cytoplasm of living cells, over a 200 nM to 5 μM concentration range. We also confirmed that an α-helical segment in the N-terminal domain contains essential determinants of Arc’s self-association.

## 1. Introduction

Arc (Activity-regulated cytoskeleton-associated protein) [1], also known as Arg 3.1 (Activity-regulated gene 3.1) [2], is an immediate early gene product that regulates multiple forms of synaptic plasticity, including long-term potentiation (LTP), long-term depression (LTD), and homeostatic plasticity [3,4,5,6]. Its role in these processes appears to be linked to its activities as a regulator of the actin cytoskeleton [3,5] and of AMAP receptor endocytosis [7]. At physiologic pH and ionic strength, Arc self-associates in vitro into multiple oligomeric species with diameters ranging from about 4 nm (monomer) to 20–35 nm (>30–40-m) [8,9,10,11]. The physiological significance of high-order Arc self-assembly has not been conclusively established, but its potential importance was highlighted by the demonstration that Arc forms virus-like particles that encapsulate mRNA for delivery into the extra-neuronal space [10,12]. Indeed, a segment comprising ~residues 207–370 in the C-terminal portion of Arc displays sequence [13] and structural [14] similarity to retroviral Gag domains. Arc dimerization and further oligomerization is driven by the second α-helix (~residues 78–140) within the N-terminal domain [15]. However, it may also be regulated by interactions with the C-terminal viral capsid-like domain, as the phosphorylation of serine 260 by Ca^2+^/calmodulin-dependent protein kinase II (CaMKII) suppresses high-order oligomerization [11].

At present, the in vivo mechanism of Arc self-assembly into virus-like structures is not understood. Arc interacts with nucleic acids in vitro and in cells [10,12]. Stripping Arc of its bound RNA prevents its assembly in vitro into ~32 nm particles that resemble viral capsids in cryo-electron micrographs [10], whereas the addition of mRNAs encoding Arc or GFP (green fluorescent protein) enhances the formation of these high-order oligomers [15]. Our previous fluorescence nanoimaging studies failed to reveal the presence of high-order oligomers in the cytosol of living cells [16]. However, these experiments were performed at average expression levels of less than 300 nM, which are considerably lower than the micromolar concentrations typically used to detect high-order Arc self-assembly in vitro. Moreover, Mergiya et al. [17] found by in situ chemical crosslinking that endogenous Arc is predominantly present as monomers and dimers in the rat brain, although low levels of trimers, tetramers, and higher-order oligomers could also be detected. Based on these observations, we have now investigated Arc oligomerization in vitro over a 1000-fold concentration range (~20 nM to ~20 μM) and performed single-point fluorescence fluctuation spectroscopy (FFS) measurements in live cells over a concentration range of ~200 nM to ~5 μM. Although we confirmed that ~30 nm diameter structures formed in vitro at micromolar concentrations, only low-order oligomers (up to tetramers) were evident in the cytosol of living cells. Using a combination of hydrodynamic and fluorescence nanoimaging approaches, we also confirmed results of Eriksen et al. [15], demonstrating the importance of an α-helix (which we term helix H2) in the N-terminal domain of Arc for its self-association in vitro and in cells. 

## 2. Results 

**Figure 1:**
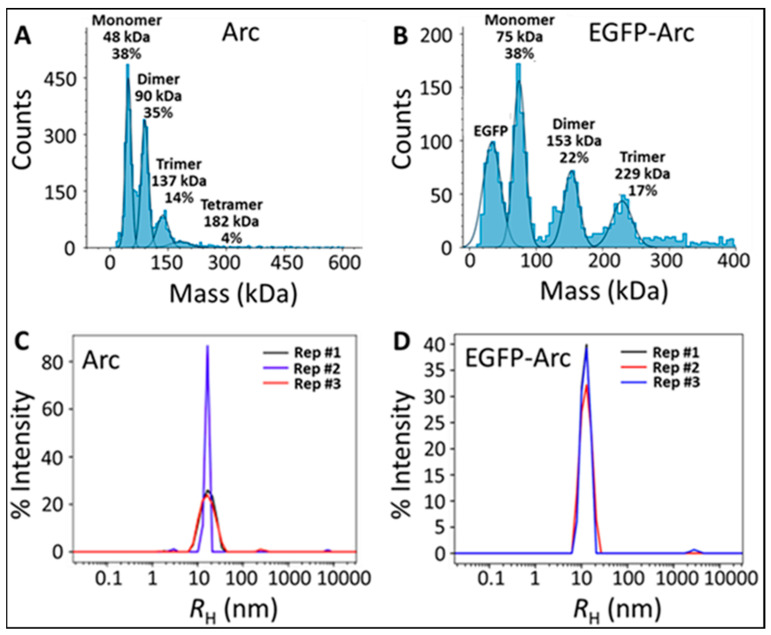
Oligomerization of bacterially expressed and purified Arc and Arc-EGFP. (**A**,**B**) Mass photometry analyses of ~20 nM Arc and EGFP-Arc in PBS. The histograms (light blue) show the number of contrast events that are related to a given molecular mass. The solid line is a Gaussian fit to the dominant peak. (**C**,**D**) Dynamic light scattering analyses of ~10 μM Arc and Arc-EGFP in 20 mM HEPES, pH 7.5, 100 mM NaCl, 0.5 mM TCEP (tris(2-carboxyethyl)phosphine), and 0.2 mM PMSF (phenylmethylsulfonyl fluoride). Three replicate measurements are shown. It is not believed that the sharpness of replicate #2 in panel C has any physical significance. All measurements were performed at 25 °C.

To date, high-order Arc oligomerization in solution has been characterized predominantly by dynamic light scattering (DLS) at Arc concentrations in the µM range [8,9,10,11]. To examine Arc self-association at lower concentrations, we used mass photometry, which has a sensitivity range of ~100 pM to ~100 nM. Mass photometry provides estimates of the molecular mass of proteins larger than ~30 kDa by quantification of the light scattered by individual particles upon binding to glass coverslips. We observed that bacterially expressed mouse Arc at ~20 nM concentration exists predominantly in a monomer/dimer/trimer equilibrium, with much lower levels of tetramers also evident (Figure 1A). Consistent with previously published values obtained by DLS and electron microscopy, the average hydrodynamic radius (R_H_) of Arc at this concentration was 17.0 ± 0.4 nm (Figure 1C). There was no evidence of low-order oligomers (monomers, dimers, etc.) in the DLS profiles. However, because the intensity of light scattered is proportional to the sixth power of the particle diameter, the contribution to the total light scattered by monomers and dimers is expected to be very small in the presence of ~30 nm diameter Arc particles.

To examine the behavior of Arc in live cells, we used fluorescence fluctuation spectroscopy (FFS), which provides estimates of the average stoichiometry from molecular brightness analysis and diffusion time determined from the autocorrelation function of fluorescently tagged proteins moving within the optical observation volume in the cell [18,19]. We first ascertained that the EGFP (enhanced GFP) tag did not interfere with low- or high-order Arc oligomerization. As with untagged Arc, mass photometry of EGFP-Arc (~20 nM final concentration) provided evidence of a monomer/dimer/trimer equilibrium (Figure 1B). Free EGFP, presumably cleaved from the fusion construct during preparation, was also evident in the profiles. We confirmed using size exclusion chromatography that the major contaminant in our Arc-EGFP samples is, indeed, free EGFP. DLS autocorrelation data revealed R_H_ distributions that were dominated by an apparently single hydrodynamic species (polydispersity index of 0.081 ± 0.018) having an R_H_ of 13.1 ± 0.4 nm (Figure 1D). Although the average R_H_ of EGFP-Arc is somewhat smaller than that of untagged Arc, the values obtained by DLS for both species are in the range of those previously reported for high-order Arc oligomers/capsids. 

**Figure 2:**
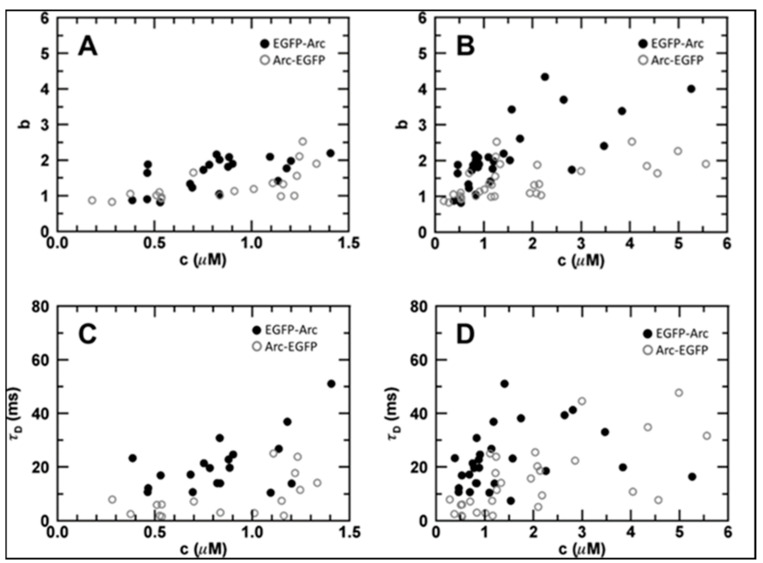
Concentration-dependent brightness and cytoplasmic diffusion of EGFP-Arc and Arc-EGFP expressed in U2OS cells. (**A**,**B**) Concentration-dependent brightness, b, of Arc with EGFP tag placed at the N-terminus (EGFP-Arc) or C-terminus (Arc-EGFP) at concentrations, c, up to 1.5 μM (**A**) or 5.5 μM (**B**). The value of b indicates the average stoichiometry of Arc in cytoplasmic complexes. (**C**,**D**) Cytoplasmic diffusion times, τ_D_, of EGFP-Arc and Arc-EGFP for concentrations up to 1.5 μM (**C**) or 5.5 μM (**D**). Each data point represents average b and τ_D_ values from a single cell (27 cells expressing Arc-EGFP and 28 cells expressing EGFP-Arc). Concentrations of EGFP-tagged proteins in each cell were estimated as described in Materials and Methods.

We performed single-point FFS analyses over a broad range of concentrations (~200 nm to ~5 µM) in U2OS cells. Brightness analyses (Figure 2A,B) indicated that cytoplasmic particles containing EGFP-tagged Arc have an average stoichiometry of 1–3 Arc molecules per particle. Autocorrelation analysis (Figure 2C,D) revealed that the diffusion times (τ_D_, which are inversely related to the diffusion coefficient, D) of EGFP-Arc and Arc-EGFP increase as a function of concentration. Soluble proteins with the molecular masses of EGFP-tagged monomeric, dimeric, and tetrameric Arc would be expected to have diffusion times of about 1.3, 1.6, and 2.0 ms, respectively. However, the measured diffusion times were scattered between ~2 and ~50 ms, confirming our previously reported observation that the diffusion process is dominated by Arc’s interaction with slowly moving partners, and not by the oligomeric state of Arc [16]. Moreover, the broad range of diffusion times suggests that these partners comprise a heterogeneous mixture of macromolecular complexes and/or transport vesicles. For comparison, τ_D_ values for β-actin mRNA [20] and small transport vesicles [21,22], as measured by FFS, are in the order of 50–100 msec. We note that the average residence times of EGFP-Arc were higher than those of Arc-EGFP, suggesting that the position of the fluorescent tag may influence the association of Arc with cytoplasmic binding partners without significantly affecting its oligomerization state.

**Figure 3:**
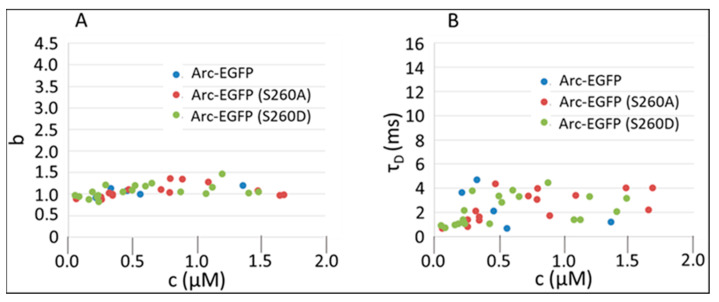
Effect of Serine 260 mutations on brightness and cytoplasmic diffusion of Arc-EGFP. (**A**) Concentration-dependent brightness, b, of C-terminally EGFP-tagged wild-type Arc and the S260A and S260D mutants. (**B**) Cytoplasmic diffusion times, τ_D_, of samples examined in panel A.

As stated, Zhang et al. [11] identified serine 260 (S260) as a site of dynamic phosphorylation by CaMKII. They showed that a phosphomimetic mutation of this site (S260D) suppressed high-order Arc oligomerization at 30 °C in vitro, whereas the S260A mutation did not affect oligomerization. To address the possibility that the absence of high-order Arc oligomers in the cell cytosol is due to its phosphorylation on serine 260, we carried out FFS analysis of Arc-EGFP (S260D) and Arc-EGFP (S260A). As shown in Figure 3, these mutations had no significant effects on the stoichiometry (Figure 3A) or residence time (Figure 3B) of Arc.

**Figure 4:**
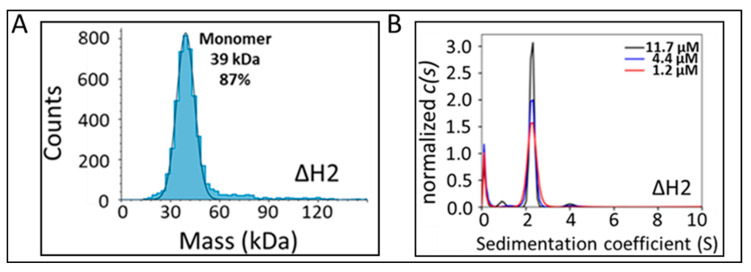
Analysis of Arc^ΔH2^ in vitro. (**A**) Mass photometry analysis of purified, recombinant Arc^ΔH2^ at 20 nM concentration. (**B**) Sedimentation velocity size distributions of three concentrations of Arc^ΔH2^. The c(s) distributions have been normalized by the areas under the respective curves. Concentrations are shown in the inset.

**An N-terminal α-helical domain is required for Arc dimerization in vitro and in cells.** Eriksen et al. [15] have identified an α-helical segment, comprising residues 99–126, as being necessary for Arc dimerization and higher-order oligomerization. Consistent with these findings, we observed that a construct lacking residues 84–132 (which we term helix H2) behaves as a monomer in solution. Mass photometry analysis of Arc^ΔH2^ at 20 nM indicated a molecular mass of ~39 kDa (Figure 4A), similar to its calculated molecular mass (including a GAMDP N-terminal extension) of 39,585 Da. Analytical ultracentrifugation (AUC) measurements carried out at Arc^ΔH2^ concentrations ranging from 1.2–11.7 μM (Figure 4B) yielded a sedimentation coefficient (S) of 2.27 ± 0.01, a molecular weight of 35,638 ± 862, and a frictional ratio (f/f_0_) of 1.67 ± 0.03. Because globular proteins have frictional ratios in the range of ~1.15–1.25 [23], we conclude that Arc^ΔH2^ is a highly asymmetric and/or flexible molecule.

**Figure 5:**
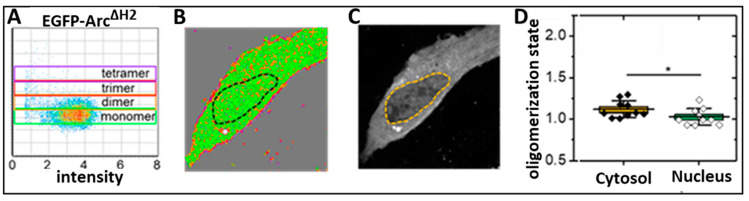
Number and Brightness (N&B) analysis of Arc^ΔH2^ in NIH-3T3 cells. (**A**) Representative brightness plots of cells expressing Arc^ΔH2^-EGFP, and EGFP-Arc^ΔH2^. Green, orange, red, and purple boxes indicate the brightness ranges of monomers, dimers, trimers, and tetramers. (**B**) Representative brightness map of the cell shown in panel C, color-coded according to the color scheme shown in panel (**A**). Green represents monomers; orange, dimers; red, trimers; and purple, higher-order oligomers. (**C**) Intensity images of the same cells. (**D**) Measured oligomerization state values (monomer = 1, dimer = 2, etc.) as a function of the intracellular protein concentration, each point representing the average of a single cell. Statistical analysis of the measured oligomerization state values using the Mann–Whitney non-parametric test (boxes: ±SE, whiskers: ±SD). * *p* < 0.05. Number of cells analyzed N = 14 (Arc^ΔH2^-EGFP) and N = 9 (EGFP-Arc^ΔH2^). Scale bar, 10 µm.

We next monitored the spatial distribution of EGFP-tagged Arc^ΔH2^ within the cell using the Number and Brightness (N&B) method [24] (Figure 5). The N&B approach differs from the molecular brightness method employed in Figure 2 and Figure 3 by providing information from each pixel and, hence, from all areas of the cell. We expressed Arc^ΔH2^-EGFP, and EGFP-Arc^ΔH2^ in NIH-3T3 cells, and representative brightness plots of single cells are shown in Figure 5A. The brightness ranges of monomers, dimers, trimers, and tetramers are indicated by green, orange, red, and purple boxes (calibrated with a solution of monomeric EGFP). Based on this color scheme, Figure 5B shows the brightness maps of representative cells, with their fluorescence intensity images shown in Figure 5C. We quantified the oligomerization state as a function of the intracellular protein concentration as shown in Figure 5D, each point representing the average of a single cell. Statistical analysis of the measured oligomerization state showed that Arc^ΔH2^ is almost exclusively monomeric in both the cell cytoplasm and nucleus.

**Figure 6:**
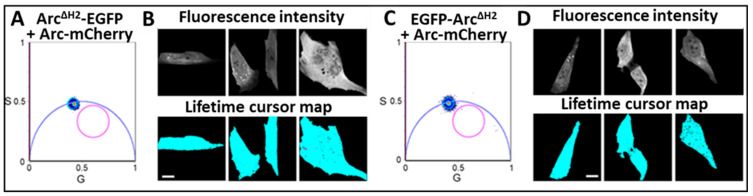
FRET/FLIM analysis in NIH-3T3 cells co-expressing EGFP-tagged Arc^ΔH2^ and mCherry-tagged Arc^WT^. (**A**,**C**) Phasor plot pixel lifetime distribution of cells co-expressing donors: Arc^ΔH2^-EGFP (panel A) or EGFP-Arc^ΔH2^ (panel C) and acceptor: Arc-mCherry. Pixels corresponding to nonquenched EGFP (i.e., not displaying FRET) are enclosed in the cyan circle. Pixels corresponding to quenched EGFP (positive for FRET) are enclosed in the magenta circle. (**B**,**D**, **top panels**) Fluorescence intensity images of cells expressing Arc^ΔH2^-EGFP (panel B) or EGFP-Arc^ΔH2^ (panel D). (**B**,**D**, **bottom panels**) Pixel lifetime mapping of cells shown in the top panels. Pixels corresponding to non-quenched EGFP are in cyan; pixels corresponding to quenched EGFP are in magenta. Absence of magenta pixels in phasor plots and lifetime cursor maps demonstrates the overall absence of FRET, as confirmed upon examination of over 100 cells. Scale bars, 10 μm.

Finally, to ascertain that Arc^ΔH2^ cannot dimerize with wild-type Arc (Arc^WT^), we measured Förster resonance energy transfer (FRET) in cells expressing Arc^ΔH2^-EGFP or EGFP-Arc^ΔH2^ together with Arc^WT^-mCherry. FRET can occur if an acceptor fluorophore, in our case mCherry, comes within a few nanometers of a donor, EGFP. Both the intensity and lifetime of the donor are reduced by energy transfer. However, because lifetime measurements are more reliable reporters of FRET in living cells, we measured FRET by reduction in the lifetime of EGFP. Using a similar approach, we had previously detected strong FRET signals in NIH-3T3 cells co-expressing Arc^WT^-EGFP and Arc^WT^-mCherry [16]. In contrast, no FRET was observed between EGFP-tagged Arc^ΔH2^ and Arc^WT^-mCherry (Figure 6). The fluorescence lifetime of each pixel was calculated and displayed using phasor plots [25]. Figure 6A,C show that the center of mass of the lifetime distributions fall on the universal semicircle, indicating a single exponential lifetime decay, as expected for unquenched EGFP. In Figure 6B,D (bottom row of panels), these pixels are highlighted with a cyan color, showing their uniform distribution within the entire cell. Almost no quenched EGFP was detected, as demonstrated by the absence of pixels highlighted with a magenta color in the cells. The corresponding fluorescence intensity images for EGFP-Arc^ΔH2^ are shown in Figure 6B,D (top row of panels). These results independently confirm that the H2 domain is critical for Arc dimerization in cells.

## 3. Discussion

Our mass photometry and DLS measurements confirmed that Arc self-associates in vitro into monomers, dimers, and trimers at ~20 nM concentration and to high-order oligomers (~30 nm diameter) at ~10 μM concentration. However, we failed to detect Arc species larger than tetramers in the cytosol of living cells by FFS, at expression levels ranging from ~0.2 to ~5.0 μM. It should be noted that our FFS approaches rely on the movement of fluorescent particles. Hence, only mobile molecules are monitored. The absence of high-order oligomers in the cytosol suggests that Arc assembles into virus-like particles on the plasma membrane or on the surface of slowly moving organelles, such as endosomes. This mechanism would not be surprising, as Gag binding to the plasma membrane (PM) is an essential step in the assembly of HIV-1 viral particles in cells [26]. In this case, the viral lattice grows by recruitment of Gag monomers and dimers from the cytosol, eventually inducing negative membrane curvature as a prelude to viral budding. Non-specific interactions between RNA and a highly basic region in the Gag N-terminal domain facilitates selective Gag binding to phosphatidylinositol (4,5)-bisphosphate on the PM. Interestingly, The N-terminal basic region of Arc also binds non-specifically to RNA [10,15]. Moreover, we have shown that Arc induces negative membrane curvature in giant unilamellar vesicles [27]. 

Recently, Eriksen and Bramham proposed an “oligomeric state hypothesis” to explain how Arc promotes such diverse neuronal activities as LTP, LTD, and intercellular communication [28]. They suggested that distinct Arc functions are driven by Arc in different oligomeric states. For example, dimers may regulate the actin cytoskeleton during LTP, whereas tetramers may promote AMPAR endocytosis during LTD. The role of high-order oligomerization (30–40 Arc subunits) is less clear. As stated, these 30–40-m may represent stages in the formation of virus-like particles. However, the reduction in synaptic depression caused by expression of the Arc (S260D) mutant, which can tetramerize but fails to form 32-m, suggests that high-order Arc oligomerization may also contribute to full expression of LTD [11]. As pointed out by Eriksen and Bramham [28], a 32-subunit “octamer of tetramers” would facilitate the putative scaffolding of endocytic proteins (e.g., dynamins and endophilins) on the clathrin-coated pit. 

Our results raise two major questions and a challenge. The first question: why can Arc self-associate into high-order oligomers in vitro at μM concentrations, whereas only low-order oligomers are evident at similar concentrations in the cytosol of living cells? Arc is an interaction hub that binds to at least 30 partners [28,29]. Therefore, it is possible that Arc associates with molecules in the cytosol that suppress its high-order self-assembly. Indeed, our autocorrelation analyses indicate that Arc monomers and dimers are present in moving cytosolic complexes that are larger than expected for proteins with the monomeric molecular masses of Arc (~45 kDa). The second, related, question raised by our studies regards the identity of the molecules that associate with Arc in the cytosol and slow its migration in FFS experiments. Many of the validated Arc interactors are membrane-associated proteins and/or are preferentially expressed in neurons. However, several others, e.g., endophilin 2, dynamin 2, and CaMKII, can also be found in the cytosol of nearly all cells. We believe that dual-color FFS approaches in cells co-expressing EGFP-tagged Arc and mCherry-tagged candidate binding partners will be useful in addressing this question. Finally, a central challenge will be to directly test the assumption that Arc assembles into high-order oligomers on biological membranes. Again, FFS approaches, perhaps coupled with total internal reflection fluorescence (TIRF), should prove useful in tackling this difficult problem.

## 4. Materials and Methods

### 4.1. Materials

Resins: Ni^2+^-NTA resin (Roche, Indianapolis, IN, USA); Pierce Glutathione Agarose (ThermoFisher Scientific, Waltham, MA, USA); Q-Sepharose (Sigma-Aldrich, Burlington, MA, USA). His_6_-maltose-binding protein (MBP)-tagged Tobacco Etch Virus (TEV) protease (Elizabeth Goldsmith, UT Southwestern Medical Center). Mouse Arc cDNA, cloning reagents, and reagents for mutagenesis were from Thermo Scientific. Primers were from Invitrogen. pGST-parallel1 vector was a gift from Hong Zhang, UT Southwestern. Reagents for electrophoresis were from Bio-Rad (Hercules, CA, USA). HEPES, Tris, protease inhibitor cocktail (cOmplate CO-RO), lysozyme, glutaraldehyde, Triton X-100, and other reagents were from Sigma-Aldrich. 

### 4.2. Cell Culture and Transfection

For brightness and autocorrelation analysis, U2OS cells (ATCC) were cultured in 10% fetal bovine serum (JR Scientific, Woodland, CA, USA) and DMEM and transfected 24 h before each measurement using GenJet In Vitro DNA Transfection Reagent (SignaGen Laboratories, Frederick, MD, USA). For Number and Brightness (N&B) and fluorescence lifetime imaging (FLIM) analysis, NIH-3T3 cells (ATCC) were plated on fibronectin-coated dishes, cultured in DMEM (supplemented with 10% FBS and 1% Pen-Strep), and transfected with Lipofectamine 3000 (ThermoFisher Scientific).

### 4.3. Constructs for Expression of Recombinant Wild-Type (WT) and Mutant Arc

To obtain GST-Arc with a TEV cleavage site (ENLYFQ) at the N terminus, mouse Arc cDNA was cloned into the pGST-parallel1 vector. This construct was also used as a template for making the ΔH2 deletion construct by PCR. To generate EGFP-tagged Arc for mammalian expression, GST-Arc was used as a template for subcloning Arc into vector pEGFP-N1 (to obtain Arc-EGFP) or pEGFP-C1 (to obtain EGFP-Arc). All DNA constructs were verified by sequencing. The His_6_-EGFP-Arc construct for bacterial expression had the following sequence: MSYY-(His_6_)-DYDIPTTENLYFQGAMDP-(EGFP)-SGLRSRA-(Arc). The resultant protein has a calculated mw of 76,368 and a theoretical isoelectric point of 5.54.

### 4.4. Protein Purification

Arc and Arc deletion/truncation mutants were expressed in *E. coli* Rosetta 2 cells and purified as previously described [9]. Cells were harvested after growing for 20 h at 16 °C. His_6_-Arc or GST-Arc was extracted from the bacterial pellet with solution A (20 mM HEPES, pH 8.0, 100 mM NaCl, 5 mM DTT, protease inhibitor cocktail, and 0.2 mM phenylmethylsulfonyl fluoride (PMSF)) and 0.05 mg/mL lysozyme. The extract was centrifuged at 100,000× *g* for 1 h and the supernatant was incubated either with Ni^2+^-NTA resin (for His_6_-Arc) or glutathione resin (for GST-Arc) for at least 4 h at 4 °C. Details of His_6_-Arc preparation were described previously [9]. Extracts containing GST-Arc were incubated with glutathione resin either directly or after precipitation of Arc with ammonium sulfate at 35% saturation. The resin was first washed with solution A, then with solution A containing 0.2% Triton X-100, and finally with solution A containing 2 M NaCl (without detergent). Washed resin was eluted either with glutathione to obtain GST-Arc or incubated with TEV protease to release Arc. Arc was dialyzed against solution B (20 mM HEPES, pH 7.5, 100 mM NaCl, 0.5 mM TCEP (reducing agent (tris(2-carboxyethyl)phosphine), and 0.2 mM PMSF. If further purification was necessary, samples were subjected to anion-exchange chromatography on Q-Sepharose. His_6_-tagged TEV was removed by incubation of Arc samples with Ni^2+^-NTA. Purified Arc was aliquoted and stored at −70 °C. To remove aggregates that formed during storage, proteins were centrifuged at 230,000× *g* for 15 min at 4 °C immediately before each experiment.

### 4.5. Analytical Ultracentrifugation (AUC)

Sedimentation velocity (SV) measurements were performed using a Beckman-Coulter Optima XL-I centrifuge. Protein samples in solution B were incubated overnight at 4 °C and introduced into the sample sectors of standard dual-sectored charcoal-filled Epon centerpieces that had been sandwiched between two sapphire windows in a cylindrical housing [29]. The reference sectors were filled with a matched buffer. The assembled centrifugation cells were inserted into an An50-Ti rotor and allowed to equilibrate at the experimental temperature (20 °C) for ~2.5 h. Centrifugation was performed at 50,000 rpm, and the time-dependent protein-concentration profiles were measured using absorbance optics tuned to 280 nm. The SV data were time-stamp-corrected [30] using REDATE (https://www.utsouthwestern.edu/research/core-facilities/mbr/software/, accessed on 5 June 2024) Corrected data were analyzed using the *c*(*s*) methodology in SEDFIT [31,32] at a resolution of 150, and maximum-entropy regularization with a confidence level of 0.683. SEDNTERP [33] was used to calculate partial-specific volumes, solution densities, and solution viscosities. Figures were rendered in GUSSI [34].

### 4.6. Mass Photometry

Oligomerization of Arc and EGFP-Arc at low concentrations was analyzed by mass photometry on a Refeyn TwoMP instrument (Refeyn, Ltd., Oxford, UK). Purified proteins in solution B were diluted to working concentrations of 200 nM in phosphate-buffered saline (PBS). Cover slips were washed with isopropanol and ultrapure water and fitted with a grid of silicon sample gaskets. Mass photometry optics were focused onto a 16.2 µL PBS droplet, and 1.8 µL of diluted sample was mixed into the droplet immediately prior to initiating a 60 s interferometric recording. This resulted in a final protein concentration of 20 nM during data collection. Data were imported into DiscoverMP (Refeyn) software (https://www.refeyn.com, accessed on 5 June 2024) and converted to ratiometric contrasts. A linear standard curve between mass and ratiometric contrast (R2 = 0.9996) was derived from BSA, thyroglobulin, and IgM standards. Automatic Gaussian curve fitting was performed in DiscoverMP software to identify peak centers and distributions. 

### 4.7. Dynamic Light Scattering (DLS)

DLS experiments were carried out in a Dynapro Nanostar instrument (Wyatt Technologies, Goleta, CA, USA) at 25 °C in buffer containing solution B. Samples (5 μL at approximately 20 µM) were dispensed into a quartz cuvette and irradiated with visible light at 661 nm. Autocorrelation data were acquired by averaging ten autocorrelation functions that had been collected for 5 s each. Three technical replicates were acquired. Data were analyzed using Dynamics, used in the regularization mode to calculate hydrodynamic radius distributions. Figures were rendered in GUSSI [34].

### 4.8. Fluorescence Fluctuation Spectroscopy (FFS)

For brightness and autocorrelation analysis (Figure 2), U2OS cells were transfected 24 h before each measurement. The beam of a Ti:Sapphire laser (Spectra Physics, Mai Tai, Mountain View, CA, USA) was focused at an excitation wavelength of 1000 nm with a 63x C-Apochromat water immersion objective (Zeiss, Oberkochen, Germany) into the cytoplasm of the cells to create a small excitation volume. EGFP-labeled Arc diffusing through the observation volume produced fluctuations in the fluorescence intensity that were recorded (Correlator.com, Bridgewater, NJ, USA) with a frequency of 20 kHz for 60 s per measurement. FFS data were analyzed using the generalized Q-parameter algorithm [35] to determine the brightness, λ, of the sample. Separate U2OS cells were transfected with EGFP alone to determine the reference brightness, λ_EGFP_, of the label by averaging the results obtained on 10 different cells. The normalized brightness, *b*, is given by the ratio of the sample to the reference brightness, *b* = λ/λ_EGFP_. Brightness, *b*, is a measure of the average oligomeric state of the sample with *b* = 1 indicating a monomeric and *b* = 2 a dimeric EGFP-labeled protein [36]. Brightness was corrected for the finite thickness of the cytoplasm by using z-scan [37]. The volume, *V*_PSF_ = 0.16 fl, and beam waist, *w*_0_ = 0.4 μm, of the two-photon point spread function were determined as previously described [37]. The molar concentration of EGFP-labeled Arc was directly calculated from the average fluorescence intensity, *F*, brightness, and PSF volume. In addition to brightness, the autocorrelation function (ACF) of the sample was calculated from the FFS data. The experimental ACF function of Arc was broadened compared to the expected ACF of a single diffusion species, indicating the presence of a heterogeneous mixture of diffusion times. To account for this broadening, we fit the data using an anomalous diffusion model [38] to determine the effective diffusion time, *τ*_D_. We also computed *τ*_D_ used a smoothing and interpolation algorithm [39,40]. Both methods yielded identical results within experimental uncertainty. *τ*_D_ is related to the diffusion coefficient, D, according to *τ*_D_ = *w*_0_^2^/4D. 

### 4.9. Number and Brightness (N&B) Analysis

NIH-3T3 cells were imaged at room temperature 15–25 h after transfection. Per data set, 100 frames of 256 × 256 pixels were acquired with a pixel dwell time of 10 μs; sample pixel size was 165 nm. For calibration, a solution of monomeric EGFP (500 nM) was prepared in PBS buffer. The true molecular brightness of EGFP monomers was measured as *b* = 0.11. All instrument parameters were kept identical. Data were analyzed in SimFCS (Globals Software, Irvine, CA, USA) (https//www.lfd.uci.edu/globals/, accessed on 5 June 2024).

### 4.10. Förster Resonance Energy Transfer/Fluorescence Lifetime Imaging (FRET/FLIM)

NIH-3T3 cells were transfected with plasmids encoding Arc^ΔH2^-EGFP or EGFP-Arc^ΔH2^, either alone or together with Arc-mCherry. Cells were imaged for a maximum duration of 90 min at room temperature 15–25 h after transfection using a Zeiss LSM880 laser scanning microscope (Carl Zeiss Inc., White Plains, NY, USA). EGFP fluorescence was excited at 880 nm (two-photon excitation, 80 MHz) and detected at 510–560 nm with a 40×, NA 1.2 water immersion lens in nondescanned mode using a hybrid photomultiplier detector (HPM-100, Becker & Hickl, Berlin, Germany) coupled to a FLIMBox (ISS, Champaign, IL, USA). Before FLIM, the presence of both donor (EGFP) and acceptor (mCherry) was verified with 488 and 594 nm excitation. For each data set, 35 frames of 256 × 256 pixels were acquired with a pixel dwell time of 16 μs. Data were analyzed in SimFCS.

### 4.11. Other Methods

Protein concentration was determined using the modified Lowry method [41] according to Peterson [42] with BSA as a standard. SDS-PAGE was carried out according to Laemmli [43]. Gels were scanned using the LICOR Odyssey system. 

## Data Availability

The original contributions presented in the study are included in the article, further inquiries can be directed to the corresponding authors.
